# Consequences of predictable temporal structure in multi-task situations

**DOI:** 10.1016/j.cognition.2022.105156

**Published:** 2022-08

**Authors:** Daniela Gresch, Sage E.P. Boettcher, Anna C. Nobre, Freek van Ede

**Affiliations:** a Department of Experimental Psychology, University of Oxford, Oxford, UK; b Oxford Centre for Human Brain Activity, Wellcome Centre for Integrative Neuroimaging, Department of Psychiatry, University of Oxford, Oxford, UK; c Institute for Brain and Behavior Amsterdam, Department of Experimental and Applied Psychology, Vrije Universiteit Amsterdam, Netherlands

**Keywords:** Working memory, Temporal expectation, Intervening task, Attention

## Abstract

In everyday life, we often anticipate the timing of an upcoming task or event while actively engaging in another. Here, we investigated the effects of predictable temporal structure within such a multi-task scenario. In a visual working-memory task, we manipulated whether the onset of a working-memory probe could be predicted in time, while also embedding a simple intervening task within the delay period. We first show that working-memory performance benefitted from temporal expectations, even though an intervening task had to be completed in the interim. Moreover, temporal predictions regarding the upcoming working-memory probe additionally affected performance on the intervening task, resulting in faster responses when the memory probe was expected early, and slower responses when the memory probe was expected late, as compared to when it was temporally unpredictable. Because the intervening task always occurred at the same time during the memory delay, differences in performance on this intervening task result from a between-task consequence of temporal expectation. Thus, we show that within multi-task settings, knowing when working-memory contents will be required for an upcoming task not only facilitates performance of the associated working-memory task, but can also influence the performance of other, intervening tasks.

## 1 Introduction

In a dynamically unfolding world, regularities help us anticipate future states of the environment and thereby guide adaptive behaviour. One potent source of such anticipation is provided by predictable temporal structures (for review see Nobre & van Ede, 2018). Resulting temporal expectations can help prepare for and optimise forthcoming perception and action (Breska & Deouell, 2014; J. T. Coull & Nobre, 1998; Denison, Heeger, & Carrasco, 2017; Heideman et al., 2018; Jones & Boltz, 1989; Praamstra, Kourtis, Hoi, & Oostenveld, 2006; Rohenkohl, Cravo, Wyart, & Nobre, 2012; Shalev, Nobre, & van Ede, 2019; Shin & Ivry, 2002; van Ede, Rohenkohl, Gould and Nobre, 2020; van Elswijk, Kleine, Overeem and Stegeman, 2007; Vangkilde, Coull, & Bundesen, 2012). Furthermore, temporal expectations can also guide prioritisation and access to working-memory contents at moments when they are most relevant (Jin, Nobre, & van Ede, 2020; Olmos-Solis, van Loon, Los, & Olivers, 2017; van Ede, Niklaus and Nobre, 2017; Zokaei, Board, Manohar, & Nobre, 2019).

To date, the effects of temporal expectation have tended to be studied within single-task contexts – that is, in the absence of intervening-task demands during the period of anticipation. This fails to capture the important ‘multi-task situation’ faced during everyday activities, in which we are often required to juggle various tasks across a common time period. In these multi-task scenarios, we may be able to anticipate the timing of an upcoming event for one task (task A), while concurrently having to engage in another task (task B). Here, we investigate whether having a predictable temporal structure for task A (here, a working-memory task) may influence performance on task B (here, a simple intervening task) in such a multi-task situation. We first ask whether the performance of a working-memory task still benefits from temporal expectations when an intervening task occurs within the period of anticipation. We additionally examine whether a predictable temporal structure for task A may also influence performance of the intervening task B, thereby affecting overall performance when juggling contemporaneous tasks.

To address these questions, we inserted an intervening task within the retention period of a visual working-memory task. Between blocks, the duration of the retention interval was either predictable (100% certainty to be short or long) or unpredictable (50% short, 50% long). Critically, the temporal-expectation manipulation only applied to the time when the items in working memory would be probed for report. In contrast, the intervening task always occurred at the same time after memory encoding, irrespective of the expected memory-probe time. Accordingly, any effects on the performance in the intervening task would reflect a between-task consequence of temporal expectations. Our primary aim in the current study was to demonstrate proof-of-concept as to whether any such between-task effects of predictable temporal structures exist.

## 2 Methods

### 2.1 Participants

The desired sample size was set to *n* = 54, building on a previous online study from our lab that targeted a complementary question using a similar overall task setup (Gresch, Boettcher, van Ede, & Nobre, 2021). To yield the targeted number of participants, we collected data from 75 online participants in total. Data from 20 participants were excluded following our a-priori trial-removal procedure before splitting data by conditions. One additional participant was also removed for using a non-memory-based strategy to complete the task (see ‘Analysis’ for details). Out of 54 participants (age range: 19 to 40; mean age: 28.37; 15 females; 38 males, 1 non-binary), 45 self-reported to be right-handed (9 left-handed).

Participants were recruited via Prolific Academic (https://www.prolific.co/) and pre-screened based on demographic criteria (age range 18 to 40, fluent in English), general health (normal or corrected-to-normal vision, no history of mental illnesses), and previous participation history on Prolific Academic (participated in at least 10 studies, with a study approval rate above 90%). All participants provided informed consent prior to participation and were paid £7.50 per hour. An additional monetary reward of up to £5 could be earned depending on participants' task performance in the memory task. Specifically, performance above 80% received a bonus payment scaling from £0.01 at 80% to £5 at 100%, with an average bonus payment of £0.67 (*SD* = 0.85) across all participants. The study was approved by the Central University Research Ethics Committee of the University of Oxford.

### 2.2 Task and procedure

**Fig. 1:**
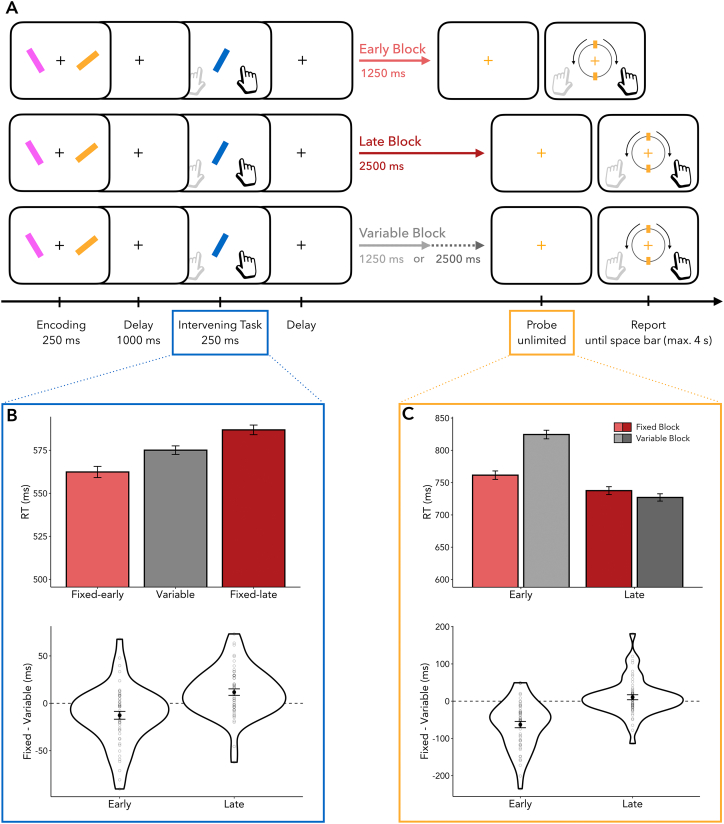
Task schematic and effects of temporal prediction on reaction times. (A) Two lateralised oriented bars were presented for 250 ms. For the working-memory task, participants had to remember the angle of both bars, of which one had to be reported at the end of the trial. Following a delay of 1000 ms, an intervening task occurred wherein another tilted bar was presented centrally for 250 ms. For this intervening task, participants had to indicate as quickly as possible whether this bar was tilted to the left or to the right. In “Early Blocks”, the intervening item offset was followed by a delay of 1250 ms, whereas in “Late Blocks”, it was followed by a delay of 2500 ms. In “Variable Blocks”, the delay between the intervening-item offset and the memory-probe onset was either 1250 ms or 2500 ms equally often. After this delay, a colour change of the central fixation cross indicated which of the two bars from the encoding display would have to be reproduced from memory. (B) Intervening task performance. Top panel: Reaction times (RTs) in the intervening task significantly increased from fixed-early to variable and from variable to fixed-late blocks. Bottom panel: Between-task effects of temporal expectation in fixed-early and fixed-late blocks, relative to variable blocks. (C) Working-memory task performance. Top panel: RTs to early probes were faster when the probe could be temporally predicted as compared to when it was temporally unpredictable. Bottom panel: Temporal expectations reduced RTs in the early condition. Violin plots for each delay condition were calculated by taking the difference in RTs between fixed variable blocks (Fixed - Variable). Dots represent individual participants. Error bars indicate ±1 standard error of the mean.

Participants performed a web-based visual working-memory task requiring them first to memorise the angles of two oriented bars and then, when probed after a retention interval, to reproduce the exact angle of one of those memory items (as in Gresch et al., 2021). In addition, participants had to perform an intervening task between encoding the memory array and retrieving the probed item (Fig. 1A). Two critical experimental manipulations concerned the time at which the memory probe appeared. Firstly, the probe could occur either after either a short (1250 ms) or long (2500 ms) retention interval (delay condition: early vs. late). Secondly, in fixed (predictable) blocks, the memory probe would always occur at the same time, whereas in variable (unpredictable) blocks, it would occur unpredictably (block type: fixed vs. variable). This resulted in four possible trial conditions: an early-fixed, early-variable, late-fixed, or late-variable onset of the memory probe. Critically, the intervening event always occurred at the same time after the disappearance of the initial array (1000 ms), independent from the memory-probe time.

Participants completed the experiment in a web browser on their personal computers. The recommended internet browsers were Mozilla Firefox and Google Chrome. Participating via mobile phone or tablet was not allowed. Prior to the experiment, participants' individual screen resolution was estimated by asking them to adjust an image of a credit card such that it matched the size of a physical credit card placed on the screen. In this manner, we could calculate the ratio between the card image width in pixels and the actual card width in centimetres to obtain a measure of pixel density (pixel per cm). Together with the instructed viewing distance of approximately 60 cm (one arm's length away from the monitor), this allowed us to present stimuli in degrees of visual angle, regardless of monitor size (Li, Joo, Yeatman, & Reinecke, 2020). The experimental script was generated in PsychoPy (Peirce et al., 2019) and hosted online using Pavlovia (https://pavlovia.org/). The experimental code can be found here, https://osf.io/rx7yv/.

At the start of each trial, two tilted bars were simultaneously presented against a grey background (RGB value: [128,128,128]) for 250 ms. One bar was always positioned to the left and the other to the right of the central fixation cross. Independent of location, one of the bars was tilted to the left (anticlockwise) and the other to the right (clockwise). To avoid angles too close to vertical or horizontal meridians, the items' angles were randomly drawn in increments of 5° between ±5° and ± 85°. Across trials, a leftward or rightward oriented bar was equally likely to appear in the left or right position of the screen. The stimuli were approximately 0.8° in width and 5.7° in length and were centred at a viewing distance of approximately 5.7° visual angle from fixation. At encoding, both lateralised items were equally likely to be probed, rendering them equally relevant.

Array disappearance was followed by a delay period of 1000 ms, during which the fixation cross remained on the screen. After this delay, the ‘intervening item’ (a tilted bar) was presented in the centre of the screen for 250 ms. Participants were required to respond to the intervening item by pressing the F key with their left index finger if the bar was tilted to the left, or the J key with their right index finger if the bar was tilted to the right.

Within blocks, the tilt of the intervening items was pseudo-randomised such that a leftward or rightward tilt occurred equally often. The intervening item was always presented in a different colour than the two memory items that preceded it. The colour of the memory items and the intervening item were always drawn from a set of three highly distinguishable colours (RGB values: blue [0,225,228], orange [254,163,0], pink [253,142,253]). The colours used for the memory items and the intervening item varied randomly across trials. The intervening item had the same size as the memory items and its angle was also randomly drawn between ±5° and ± 85° in increments of 5°.

The offset of the intervening item was followed by a second delay period (1250 ms or 2500 ms) before the fixation cross changed to match the colour of one of the two memory items, to indicate which memorised tilt should be reported. Importantly, the colour of the probe would never match that of the intervening item. In fixed blocks, the second delay interval had a fixed duration of 1250 ms (early blocks) or 2500 ms (late blocks) across all trials. In variable blocks, half of the trials had a retention interval of 1250 ms, whereas the other half had a memory delay of 2500 ms. The order of trials within variable blocks was randomised.

For the working-memory task, participants had to try to reproduce the exact angle of the probed memory item. As such, in contrast to the intervening task requiring simple discrimination (leftwards or rightwards), the memory task demanded a precision response. Following the appearance of the probe, participants had unlimited time to decide on their response. After response initiation, a visual response dial was displayed on the screen, always starting in the vertical position. The response dial included markers along a circle that corresponded to the ends of a bar and always appeared surrounding the fixation.

To report a leftward (rightward) angle, participants were (as for the intervening task described above) asked to press the F or J key on the keyboard using their left or right index finger. The dial rotated leftwards when pressing F and rightwards when pressing J (either holding the key down or pressing the key repeatedly; always in increments of 5°). The dial could only be rotated in the direction that was initially indicated by the participant. For example, if a participant started pressing the F key after the probe, the dial would only move leftwards, and it would therefore not be possible to move the dial towards the right with the J key. Since the response dial always started in the vertical position and because it could not be rotated beyond ±90°, a leftward (or rightward) oriented bar could only be correctly reported with the left (or right) key. Consequently, the hand required for responding was directly linked to the angle of the bar that was probed. This builds on previous tasks from our lab (Boettcher, Gresch, Nobre, & van Ede, 2021; Gresch et al., 2021; van Ede, Chekroud, Stokes and Nobre, 2019), though we note that the specifics of this response implementation were not essential to the current study. Once participants started rotating the dial, they had limited time (4000 ms) to complete the angle reproduction. This was intended to encourage participants to recall the exact orientation before starting to move the dial. When the dial aligned with the remembered tilt of the item, participants pressed the space bar to confirm their response and continue with the task.

At the end of each trial, participants received feedback about their working-memory performance and, when relevant, also about their intervening-task performance. The working-memory feedback provided information on how well participants reproduced the probed item. Feedback was presented for 500 ms in the form of a number ranging from 0 to 100, with 100 indicating a perfect report and 0 indicating that the adjusted orientation was perpendicular to the angle of the probed item. However, if the time to adjust the angle ran out, the message ‘Too slow’ was presented instead for 750 ms. Additional feedback could also appear to indicate if participants responded with the wrong key or did not respond at all to the intervening item. To incentivise fast responses in the intervening task, participants also received a feedback message when their reaction time (RT) was slower than 1000 ms. This feedback message was combined with an image reminding participants to press F (or J) when the intervening item was tilted to the left (or right). Feedback was presented for a minimum of 750 ms and until the space key was pressed to encourage participants to read the feedback message before being able to continue to the next trial. Trials were separated by an intertrial interval randomly drawn between 500 and 800 ms.

The experiment consisted of 384 trials divided across 12 blocks (each including 32 trials). In six blocks, the delay between intervening-item offset and probe onset was fixed (predictable) – with the probe only occurring early in three of the blocks, and only occurring late in the other three. In the remaining six blocks, probe onset was variable (unpredictable; pseudo-randomly varying between early and late within each block). As such, the total number of trials in which the probe appeared at any one delay interval after offset of the intervening item (early vs. late) was equal across the fixed and variable blocks. The order of blocks was pseudo-randomised in groups of four containing two variable blocks, one fixed-early, and one fixed-late block.

To become familiarised with the procedure of the experiment, participants performed 32 practice trials each with an unpredictable delay period. Participants were informed that they would never have to reproduce the tilt of the intervening item. However, they were not informed about the block type (fixed vs. variable) or the two possible probe-onset times (early vs. late). The instructions also stressed that for both the intervening and working-memory task, participants should respond as quickly and accurately as possible. At the end of the experiment, participants were redirected to the survey website Qualtrics (http://www.qualtrics.com/), where they were asked about their comprehension of the instructions, potential strategies used to complete the task, and whether or not their data could be analysed. The experiment lasted approximately 50 min in its entirety.

### 2.3 Analysis

Data were analysed in R Studio (RStudio Team, 2019). During pre-processing, trials were removed when RTs in the working-memory task (calculated from probe onset to response onset) were below 200 ms or exceeded 5000 ms. Next, we removed trials for which the remaining RTs were 2.5 *SD* above the individual mean across all conditions or if participants took longer than 4000 ms to reproduce the probed angle after response initiation. Regarding the intervening task, we excluded trials if participants either did not respond at all or if they did not respond within a time window ranging from 200 ms to 1500 ms after the intervening-task onset. Datasets with more than 25% of trials rejected during these pre-processing steps or with average reproduction errors higher than 40° in the working-memory task (across all conditions) were removed from further analysis (*n* = 20). Additionally, one dataset was also removed in which the participant self-reported as having employed an explicit non-memory-based strategy to maintain the encoding display (e.g., physically aligning their fingers with the memory items at encoding). After this exclusion step, datasets from the remaining 54 participants (in which an average of 95.18% [*SD* = 2.78] of trials were retained) were entered into the main analysis. Detailed information regarding the removal of trials per participant can be found in the analysis script. The analysis script and data can be found here, https://osf.io/rx7yv/.

For the working-memory task, we examined the average RTs for the conditions fixed-early, variable-early, fixed-late, and variable-late. Moreover, we also evaluated reproduction errors by averaging the absolute difference between the original angle of the target (probed) item and the reported angle.

For the intervening task, we analysed the average RTs for the fixed-early, fixed-late, and the variable condition. We did not split the variable condition by early and late trials, as at time of intervening-task onset, it was unknown whether the working-memory probe would occur early or late. For the same conditions, we also calculated the average error rates. Participants committed an error when using the wrong key to respond to the intervening item. Since we expected error rates for this simple discrimination task to be quite low, RTs were deemed the more sensitive dependent variable for the intervening-task performance. To examine potential sequential effects in variable blocks, we analysed RTs and error rates as a function of the delay condition associated with the working-memory task in the previous trial (previous probe early vs. previous probe late). Unlike classic sequential effects that are considered within single-task situations (for a review see: Los, 2010), we here investigated potential sequential effects of the preceding delay of the working-memory task on performance in the intervening task that always occurred at the same time after memory encoding.

When comparing more than two means, we applied a repeated-measures analysis of variance (ANOVA) and reported η^2^_G_ as a measure of effect size. When evaluating only two means we applied a paired samples *t*-test and report Cohen's *d* as a measure of effect size. For post hoc *t*-tests, we report Bonferroni-corrected *p* values that we denote as “*p*_Bonferroni_”. We used the ggplot2 package (version 3.3.3; Wickham, 2009) for plotting results. Where relevant, the within-subject standard error of the mean was calculated from normalized data using the approach from (Morey, 2008).

## 3 Results

### 3.1 Temporal predictions improve working-memory performance

We first confirmed that our manipulation of temporal predictions for the working-memory task was effective despite an intervening task occurring in the period of anticipation. For this, we evaluated RTs, which we defined as the time between the onset of the memory probe and response initiation. RTs served as a proxy for the time it took participants to access the relevant memory information before starting to reproduce the probed angle. We found a significant main effect of delay condition and block type: RTs to the probe were faster in late as compared to early trials (*F*_(1,53)_ = 62.517, *p* < 0.001, η^2^_G_ = 0.024) and when probe onset was fixed compared to variable (*F*_(1,53)_ = 22.491, *p* < 0.001, η^2^_G_ = 0.005). These two main effects were paired with a significant interaction between the delay condition and block type (Fig. 1C; *F*_(1,53)_ = 48.396, *p* < 0.001, η^2^_G_ = 0.009). This interaction showed that temporal predictions conferred a significant benefit (i.e., led to faster response initiation) for early probes (*t*_(53)_ = −7.437, *p*_Bonferroni_ < 0.001, *d* = 1.012), but not for late probes (*t*_(53)_ = 1.568, *p*_Bonferroni_ = 0.491, *d* = 0.213). This finding is typical of studies of temporal expectation (as reviewed in Nobre & van Ede, 2018) and is attributed to the fact that, once the early interval passes, participants always know the memory will be probed at the later interval, regardless of which block they are in. Moreover, pairwise comparisons revealed that participants responded more slowly to early as compared to late memory probes in variable blocks (*t*_(53)_ = 10.633, *p*_Bonferroni_ < 0.001, *d* = 1.447), whereas the difference in RTs in early-fixed versus late-fixed blocks did not reach significance (*t*_(53)_ = 2.531, *p*_Bonferroni_ = 0.058, *d* = 0.344).

It should be noted that the response window for the intervening task (relative to memory probe onset) differed in trials with early versus late memory probes. Although we only analysed trials in which participants responded within 1500 ms after intervening-task onset, it is possible that RTs to the working-memory task were affected by the recovery time available between the intervening and memory-task responses in early as compared to late trials. To ensure that our findings could not be attributed to this difference, we reran the pre-processing and analysis while excluding slower responses to the intervening task (RTs > 1000 ms rejected, with 8.42 ± 8.80% trials removed per participant). The observed benefit of temporal prediction remained the same even when ensuring a minimum recovery time of 500 ms (Supplementary Table 1, Supplementary Fig. 1).

**Fig. 2:**
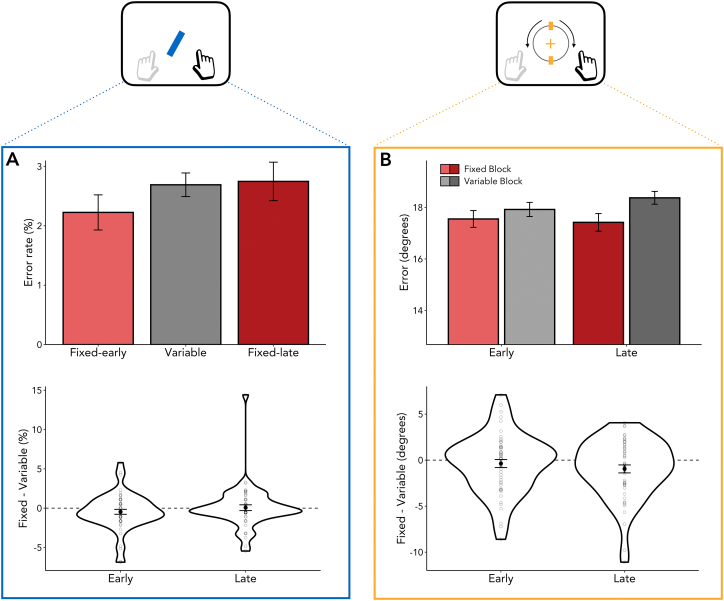
Effects of temporal prediction on reproduction errors. (A) Intervening task. Top panel: Error rates did not differ significantly between conditions. Bottom panel: Temporal expectations did not influence error rates. (B) Working-memory task. Top panel: Reproduction errors were significantly higher in variable versus fixed blocks. Bottom panel: Temporal expectations decreased error rates for both delays. Violin plots for each delay were calculated by taking the difference in error rates between fixed blocks and variable blocks (Fixed - Variable). Dots represent individual participants. Error bars indicate ±1 standard error of the mean.

Next to RTs, we additionally considered the influence of temporal predictions on the quality of working-memory reports. To this end, we analysed the average reproduction error (i.e., the absolute deviation between the reported angle and the true angle of the probed item), for which lower values indicate better performance. We observed a significant effect of block type on reproduction errors (Fig. 2B; *F*_(1,53)_ = 4.854, *p* = 0.032, η^2^_G_ = 0.002), with smaller errors when the memory probe occurred in a fixed versus a variable block. In contrast to RTs, however, for errors we found no systematic difference between early and late probes (*F*_(1,53)_ = 0.313, *p* = 0.578, η^2^_G_ < 0.001) nor an interaction between block type and delay condition (*F*_(1,53)_ = 0.865, *p* = 0.357, η^2^_G_ < 0.001).

These data reveal that temporal expectations were employed to anticipate upcoming memory-guided behaviour, building on our prior demonstrations of similar effects in the absence of intervening tasks (Jin et al., 2020; van Ede, Niklaus and Nobre, 2017; Zokaei et al., 2019). The current results show that benefits of temporal expectation on working-memory guided behaviour occur even when an intervening task must be completed during the retention interval.

### 3.2 Between-task consequences of temporal predictions for intervening-task performance

Next, we asked whether temporal expectations regarding the subsequent working-memory probe influenced performance on the intervening task. Critically, the intervening task always occurred at the same time after encoding, so any potential performative differences in this task should reflect a between-task consequence of temporal expectation regarding the working-memory task. Note that we did not divide variable blocks by the subsequent interval for the memory probe since participants could not have known whether the memory items would be probed early or late at the time of the intervening task.

As shown in Fig. 1B, responses to the intervening task were fastest when the memory probe was expected early, slowest when the memory probe was expected late, and intermediate when the memory probe occurred unpredictably. This pattern of results was supported by a significant main effect (*F*_(2,106)_ = 18.282, *p* < 0.001, η^2^_G_ = 0.007). Pairwise comparisons showed significantly faster RTs in fixed-early as compared to both the variable (*t*_(53)_ = −3.067, *p*_Bonferroni_ = 0.010, *d* = 0.417) and the fixed-late blocks (*t*_(53)_ = −5.427, *p*_Bonferroni_ < 0.001, *d* = 0.739), as well as significantly faster RTs to variable as compared to fixed-late blocks (*t*_(53)_ = −3.443, *p*_Bonferroni_ = 0.003, *d* = 0.468). These results show that temporal expectations do not merely influence performance in the task to which they apply (in our case, the working-memory task); they also affect performance in another task that occurs during the period of temporal anticipation – even when this task itself always falls within the exact same timeframe after memory encoding.

Consistent with the RTs, error rates in the intervening task (Fig. 2A) were numerically smallest in fixed-early and largest in fixed-late blocks. However, for error rates we did not find a significant difference between conditions (*F*_(2,106)_ = 1.060, *p* = 0.350, η^2^_G_ = 0.004). This may reflect intervening-task performance being very close to ceiling (error rates below 3%). Hence, we refrained from further analysing and interpreting accuracy on the intervening task.

### 3.3 Between-task sequential effects of temporal structure

After having demonstrated that temporal expectations affected intervening-task performance in fixed blocks, wherein participants are persistently exposed to the same temporal structure, we asked whether temporal associations over a short timescale can also guide performance. To this end, we analysed sequential effects in variable blocks by comparing RTs and error rates to the intervening task as a function of the working-memory delay in the immediately preceding trial (*n*-1). In contrast to classical sequential effects examined in simple RT tasks (for a review see: Los, 2010), we here tested for potential sequential effects of the previous working-memory delay on performance in the intervening task.

**Fig. 3:**
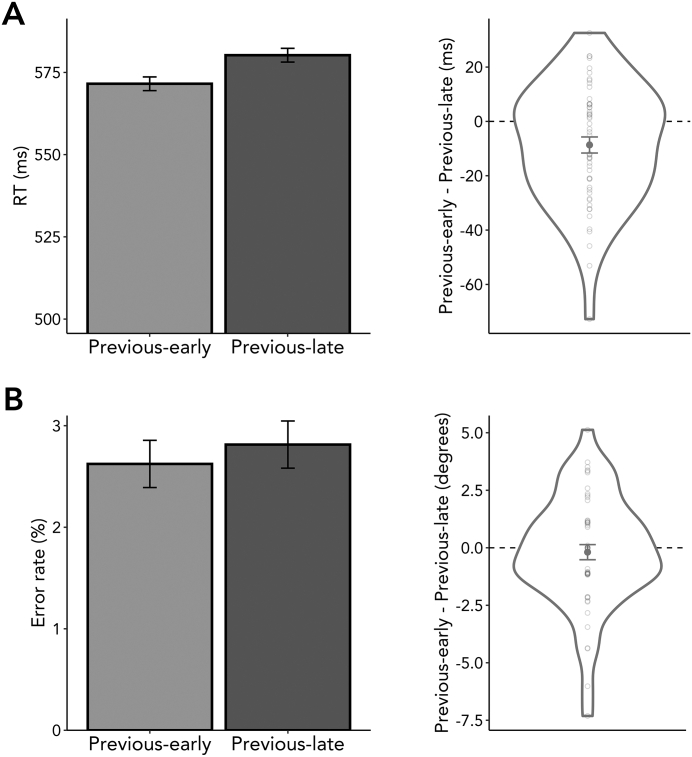
Sequential effects of temporal intervals on intervening-task performance (A) Left panel: Reaction times (RTs) in variable blocks of the intervening task were significantly faster when the immediately preceding working-memory probe occurred early versus late. Right panel: Violin plots showing the difference in RTs between trials of which the working-memory delay of the preceding trial was early versus late (Previous-early – Previous-late) (B) Left panel: Error rates in variable blocks did not differ significantly depending on the working-memory delay interval of the previous trial. Right panel: As in the right panel in Fig. 3A, with the difference in errors rates instead of RTs. Dots represent individual participants. Error bars indicate ±1 standard error of the mean.

As shown in Fig. 3A, RTs to the intervening task were faster when the working-memory probe in trial *n*-1 occurred early as compared to when it occurred late (*t*_(53)_ = −2.937, *p* = 0.005, *d* = 0.400). The temporal structure associated with the working-memory delay of the previous trial thus had an immediate effect on RTs in the intervening task of the current trial – consequently, demonstrating a sequential-effect influence on the intervening task. Error rates showed a similar numerical pattern (Fig. 3B), though pairwise comparisons did not reach significance (*t*_(53)_ = −0.580, *p* = 0.564, *d* = 0.079).

Thus, the temporal interval of the working-memory task in the preceding trial significantly affected performance of the intervening task in the current trial. Nevertheless, the effects of blocked temporal predictions were stronger (Supplementary Fig. 2A). The difference in RTs between early versus late trials in fixed blocks was significantly larger than the difference in RTs following trials with early versus late working-memory probes in variable blocks (*t*_(53)_ = −3.260, *p* = 0.002, *d* = 0.444; Supplementary Fig. 2B).

## 4 Discussion

By manipulating the predictability of temporal structures within a multi-task context, we made two relevant observations. First, temporal expectations about when to utilise working-memory contents confer significant benefits to memory performance even when other intervening tasks must be performed in the interim. Second, temporal expectations about the later working-memory task also had significant consequences for performance of the intervening task, even though the temporal prediction manipulation did not apply to this task. Responses to an intervening task were expedited when the requirement for memory-guided behaviour was expected to occur early and slowed when the working-memory task was expected late, as compared to when the timing of the working-memory task was unpredictable. Thus, we unmask between-task consequences of predictable temporal structures.

### 4.1 Between-task consequences of predictable temporal structures

Our work clearly demonstrates the presence of a between-task consequence of predictable temporal structures in a multi-task setting. It invites consideration of what mechanisms account for the effects, and this remains an important direction for future research. At this juncture, we can only speculate regarding the putative mechanisms.

First, it is possible that the between-task effect may result from more efficient ‘scheduling’ of both tasks when the timing of either of them is predictable. In line with this, Kushleyeva, Salvucci, and Lee (2005) proposed that, in multi-task scenarios, temporal aspects of each task are used to flexibly schedule when to prioritise one task or the other. Given there was consistently less time between the intervening item and memory probe in the fixed-early blocks, participants may have opted to complete the intervening task as soon as possible to refocus on the working-memory task in time for the probe. In contrast, when the working-memory probe was consistently expected late – yielding less time pressure – participants may have allowed themselves more time for the intervening task, rendering their responses slower.

Another potential interpretation is that an elevated state of preparedness or ‘alertness’ may have accompanied temporal expectations for an early working-memory probe, whereas a lowered state of alertness may have resulted when the probe was expected late (Shalev & Nobre, 2022; Weinbach & Henik, 2012). The differential overarching state of preparation or alertness may have effectively ‘spilled over’ to the intervening task. An alternative spill-over interpretation relates to a suggestion in the time-perception literature that cross-contamination between temporal intervals occurs when more than a single interval is estimated simultaneously (Moon & Anderson, 2013; Taatgen & van Rijn, 2011). Specifically, representations of shorter or longer intervals can shift the representation of an intermediate interval in those respective directions. Such a contamination effect may also have contributed to our observed between-task effect of predictable temporal structures. For example, in fixed-late trials, the representation of time used for guiding performance in the intervening task may have expanded and thereby slowed responses. Likewise, faster RTs in fixed-early trials may have been mediated through compression of time representation. Future studies should take a more granular, and complementary physiological approach to understand exactly how these factors contribute to the between-task consequence of temporal expectations that we exposed here.

In addition to the scenarios above, it may have been easier to anticipate the intervening task when the working-memory task itself was also temporally predictable. However, this is unlikely to account for our results, as this would have led to faster RTs to the intervening task regardless of whether the working-memory probe occurred early or late. In contrast, we found that it was not the temporal predictability per se that affected performance of the intervening task, but rather the time at which the subsequent probe was expected in the fixed blocks (early vs. late). Yet, it remains an interesting question whether we would observe similar results if the intervening task itself had occurred at random moments during the anticipatory period.

### 4.2 Between-task consequences of temporal structures also occur over the short-term

Aside from the between-task consequences of predictable temporal structure between fixed and variable blocks, we also found between-task short-term effects of temporal structure in variable blocks. RTs to the intervening task in variable blocks were faster when the previous working-memory probe occurred early as opposed to late. Our effects in this multi-task context provide an interesting extension to observations during variable-foreperiod tasks, in which the interval (foreperiod) between a warning signal and an imperative target varies on a trial-by-trial basis (Drazin, 1961; Karlin, 1959; Los, 1996, Los, 2010; Los, Kruijne, & Meeter, 2014; Niemi & Näätänen, 1981; Steinborn, Rolke, Bratzke, & Ulrich, 2008; Vallesi & Shallice, 2007; Woodrow, 1914). A typical finding in these variable-foreperiod tasks is the asymmetric sequential effect: RTs for any current foreperiod are relatively insensitive to preceding shorter foreperiods, however increase if the previous foreperiod was longer than the current one (for a review see: Los, 2010). Similarly, in our study the speed of responses to the intervening task may have been scaled relative to the memory-probe delay of the trial directly prior. However, direct comparisons between our results and single-task sequential effects are difficult to derive and will require further experimentation. Unlike most single perceptual-motor tasks evaluating temporal preparation, our study comprised a multi-task design engaging both internal and external attention. Moreover, each trial in our experiment contained three important intervals – the interval between encoding offset and intervening-task onset (always 1000 ms), the interval between intervening-item offset and the memory probe (early probe: 1250 ms, late probe: 2500 ms), and the ITI (randomly drawn between 500 ms and 800 ms), rendering it neither a purely fixed nor variable design. Nevertheless, it is intriguing that we observed a sequential effect on RTs in the intervening task (which always occurred at the same point in time) arising from the delay condition of the working-memory task of the previous trial.

At the same time, we note that the existence of sequential effects does not fully account for the performance benefits related to temporal predictability in fixed versus variable blocks. We show a higher difference in RTs between early and late trials in fixed blocks as opposed to previous-early versus previous-late trials in variable blocks. To the extent that implicit temporal associations underpin the effects of temporal expectation or preparation in fixed blocks, our results suggest that strengthening of evidence and learning over longer timescales leads to stronger impacts on performance. The multiple trace theory of temporal preparation (MTP) provides a good candidate mechanism, capturing the ability of temporal associations over multiple timeframes to modulate behaviour (Los et al., 2014; Los, Kruijne, & Meeter, 2017; Salet, Kruijne, van Rijn, Los, & Meeter, 2022). In the future, it will be interesting to utilise neural measures to characterise exactly how temporal regularities over the different timescales shape signals guiding temporal expectation and preparation that can operate across tasks.

### 4.3 The contribution of task relevance and temporal structure in multi-task contexts

At first glance, the beneficial consequence of predictable temporal structures in one task on performance in another task may seem to contradict findings related to the selective nature of temporal expectations. For example, previous research has demonstrated that directing attention to a target occurring at one point in time leads to performance benefits at that specific time but simultaneously to impairments earlier and later (Denison et al., 2017, Denison, Carrasco, & Heeger, 2021). Instead, we show that temporal expectations can facilitate performance of the task to which they are applied, as well as the intervening task. In reconciling these findings, it is important to note at least two key differences which may help resolve this apparent discrepancy. In Denison et al.'s (2017, 2021) study, participants performed only one task. Stimuli within that task had overlapping sensory properties and action associations, competing for priority in guiding performance. As one of the stimuli was more likely to be irrelevant, a trade-off between prioritising the likely-relevant stimulus and ignoring the likely-irrelevant stimulus was therefore an effective strategy. By contrast, a strategic trade-off would have been counterproductive in our study, as participants completed two separate tasks that were equally relevant. This provides for a scenario wherein both tasks potentially benefitting from temporal expectation would be much more advantageous.

Accordingly, our pattern of results is likely driven by the requirement of having to perform two relevant tasks. An additional intriguing question is whether the cross-task benefits of predictable temporal structure are also contingent on the specific parameters and demands of the two tasks. In our study, a choice-reaction task was embedded within a working-memory task, to which participants had to return upon completion of the intervening task. Participants therefore had to juggle the contents of stimulus representations to perform adequately. Would similar results occur if another perceptual-motor choice-reaction task replaced the working-memory task? Such a finding would suggest that regularities in temporal structure alone could explain performance benefits across tasks, even when the need to maintain mental contents necessary for one task does not overlap the timespan of another. Therefore, in future studies it will be interesting to examine the extent to which benefits of temporal structure can occur between two independent rather than superimposed tasks. Manipulating the perceptual similarity and motor requirements between tasks would add further insights about the role of overlapping demands in orchestrating behaviour across successive tasks.

### 4.4 Temporal expectations benefit working-memory guided behaviour despite intervening-task demands

Beyond providing evidence for a between-task consequence of temporal predictability, our study also yields new insights into the dynamic and prospective nature of working memory. Extending the growing literature showing the benefits of temporal expectation on working-memory performance (Boettcher, Gresch, et al., 2021; Gresch et al., 2021; Jin et al., 2020; Olmos-Solis et al., 2017; van Ede, Niklaus and Nobre, 2017; Wilsch, Henry, Herrmann, Herrmann, & Obleser, 2018; Wilsch, Henry, Herrmann, Maess, & Obleser, 2015; Zokaei et al., 2019), our results uniquely demonstrate that performance benefits of temporal expectations during working memory remain robust, even when having to perform an intervening task in the period of probe anticipation.

From the time-perception literature, there is abundant evidence that the ability to track time explicitly can be biased when performed in complex multi-task situations (Block, Hancock, & Zakay, 2010; Brown, 1997, Brown, 2006; Brown, Collier, & Night, 2013; Fortin, Champagne, & Poirier, 2007; Hemmes, Brown, & Kladopoulos, 2004; Polti, Martin, & Van Wassenhove, 2018). Potentially, having to engage in the intervening task could have impeded the utilisation of temporal regularities regarding the working-memory task. Indeed, prior studies have shown that temporal-expectation effects can be substantially reduced by performing a concurrent demanding task (Capizzi, Correa, & Sanabria, 2013; Capizzi, Sanabria, & Correa, 2012; van der Mijn and van Rijn, 2021). Yet, a carefully controlled study using dual-task designs has shown that foreperiod effects survive in the face of a simultaneously performed task (van Lambalgen and Los, 2008). In a similar vein, recent work from our lab has revealed that learned (spatio)temporal regularities regarding external events can guide visual-search performance even when interacting with temporally unpredictable intervening events (Boettcher, Shalev, Wolfe, & Nobre, 2021). Likewise, temporal expectations affect performance even during dynamic streams that require inhibition of temporally competing distractors (Chauvin, Gillebert, Rohenkohl, Humphreys, & Nobre, 2016; Davranche, Nazarian, Vidal, & Coull, 2011; Zokaei et al., 2021). These results suggest that it is possible to maintain and utilise representations of temporal regularities across other intervening events. The current results extend these findings, by demonstrating that temporal regularities can also be highly effective for facilitating working-memory-guided behaviour during multi-task situations.

Nevertheless, our ability to keep track of time might be dependent on the specific demands of the intervening tasks, whereby more cognitively taxing intervening tasks may be more detrimental to the formation of temporal expectations. Moreover, the utilisation of temporal expectations for memory-guided behaviour may be further affected by the overlap between intervening and memory items. Prior research has found evidence for working-memory performance to be most impaired when perceptual distractors are similar to the memory content (Clapp, Rubens, & Gazzaley, 2010; Hermann et al., 2021; Jha, Fabian, & Aguirre, 2004; Sreenivasan & Jha, 2007; Yoon, Curtis, & D'Esposito, 2006). Therefore, a high degree of similarity in the associated objects between tasks may modulate the benefit of temporal expectations. Within our study, additional analyses did not support this idea (Supplementary Results 1, Supplementary Table 2 and 3, Supplementary Fig. 3). Perceptual similarity between intervening and memory items did not affect RTs, and reproduction errors were in fact smaller when the intervening and memory item were more similar than when the angular difference between both items was high. We also observed no interactions between sensory similarity and either block type or delay condition, suggesting the effects of temporal expectation were not dependent on the sensory overlap. Future studies may wish to explore more systematically the possible contribution of sensory and motor overlap, as well as task difficulty between tasks.

Temporal expectations have been reported to benefit performance particularly at short as opposed to long delays. This effect is usually attributed to the hazard rate: once the potential time of the early probe passes, expectations can be updated, and attention can be reoriented towards the longer interval (Coull, 2009; Coull, Frith, Büchel, & Nobre, 2000; Griffin, Miniussi, & Nobre, 2002; Jin et al., 2020; Miniussi, Wilding, Coull, & Nobre, 1999; Nobre, 2001). Thus, even in variable blocks, the late memory probe becomes predictable as soon as the early interval has passed (see also MTP [e.g., Los, Kruijne and Meeter, 2014, Los, Kruijne and Meeter, 2017; Salet et al. (2022)] for a non-hazard explanation of this effect based on memory traces of past timing experiences). This pattern of RTs has been noted in the foreperiod literature, with shorter RTs occurring with increasing delay duration in variable-foreperiod designs. In contrast, RTs in fixed-foreperiod situations increase with increasing delay between warning and target stimulus (Los et al., 2014; Los, Knol, & Boers, 2001; Niemi & Näätänen, 1981). In the variable blocks of the current study, RTs in the working-memory task reflected this typical pattern of results – responses to the memory probe were faster when it appeared unpredictably late as compared to unpredictably early. However, we did not find an effect of delay condition on RTs in fixed blocks as would have been predicted by the foreperiod literature. This was the case even after excluding slow responses to the intervening task, thus, giving time to recover before performing the memory task. Whether this effect is specific to the multi-task context of our study is an interesting avenue for future research.

Interestingly, memory representations were more accurate in fixed as compared to variable trials, that is, even when the memory probe appeared late (see also Chauvin et al., 2016; Cravo, Rohenkohl, Santos, & Nobre, 2017). This is distinct from a previous study conducted in our lab which also examined the role of temporal expectation in working memory (Jin et al., 2020). Critically, however, in this prior study, no intervening task occurred during the working-memory delay. The intervening task in the present study may thus be critical for the here-observed effect of temporal expectations on working-memory performance in the late trials. We recently showed that working memory can be shielded against intervening tasks demands when the intervening task itself can be temporally predicted (Gresch et al., 2021). Similarly, having temporal expectations about the memory probe may have helped to shield the working-memory representations from the intervening task, yielding higher ensuing memory accuracy in fixed trials, regardless of whether items would subsequently become probed early or late.

## 5 Conclusion

In the current work, we focussed on the intersection of several lines of research often studied in isolation, including working memory, temporal expectations, and multitasking. We provide evidence (1) that learned temporal expectations regarding task A can be utilised even when having to engage in an intervening task B during the period of anticipation and (2) that temporal expectations regarding task A can affect the performance of intervening task B. Thus, the predictable temporal structure of one task facilitates not only the performance of this task but can affect and improve performance of multiple tasks in temporally structured multi-task situations.

## Appendix A Supplementary data

Supplementary material Image 1
