# Supplementary material for: Consequences of predictable temporal structure in multi-task situations
Source: Cognition. 2022 Aug;225:105156. doi: 10.1016/j.cognition.2022.105156 (PMC9760566; doi:10.1016/j.cognition.2022.105156)
Supplement: Supplementary file 1 — Supplementary material [file mmc1.pdf]

Supplementary Material

Supplementary Results 1

Working-memory performance as a function of sensory similarity, block type, and delay condition

As an additional analysis, we examined whether the observed temporal expectation effects in the working-memory task were modulated by the sensory similarity between the intervening and memory item. We calculated the absolute angular differences between the intervening item and the memory item, sorted them ordinally, and binned them into three bins. This resulted in three levels of sensory similarity, that is, low (60° to 90° angular difference), medium (30° to 60° angular difference), and high (0° to 30° angular difference). As a next step, we performed a repeated-measures ANOVA with the factors delay condition, block type, and sensory similarity.

RTs did not differ between more versus less similar items, and none of the interactions including the factor sensory similarity reached significance (Supplementary Figure 3A, see Supplementary Table 2 for the full set of inferential statistics). For reproduction errors, we found a main effect of sensory similarity ($F_{(2,106)}$ = 5.411, p = 0.006, η^2_G = 0.002, Supplementary Figure 3B). Pairwise comparisons revealed that errors were higher when the similarity between items was low ($t_{(53)}$ = -2.911, $p_{\text{Bonferroni}}$ = 0.016, *d* = 0.396) or medium ($t_{(53)}$ = -3.150, $p_{\text{Bonferroni}}$ = 0.008, *d* = 0.429) as compared to high. However, there was no difference in errors between trials with low and medium sensory similarity ($t_{(53)}$ = -0.515, $p_{\text{Bonferroni}}$ = 1.000, *d* = 0.070). As for RTs, none of the interactions including the factor sensory similarity reached significance (see Supplementary Table 3 for the full set of inferential statistics).

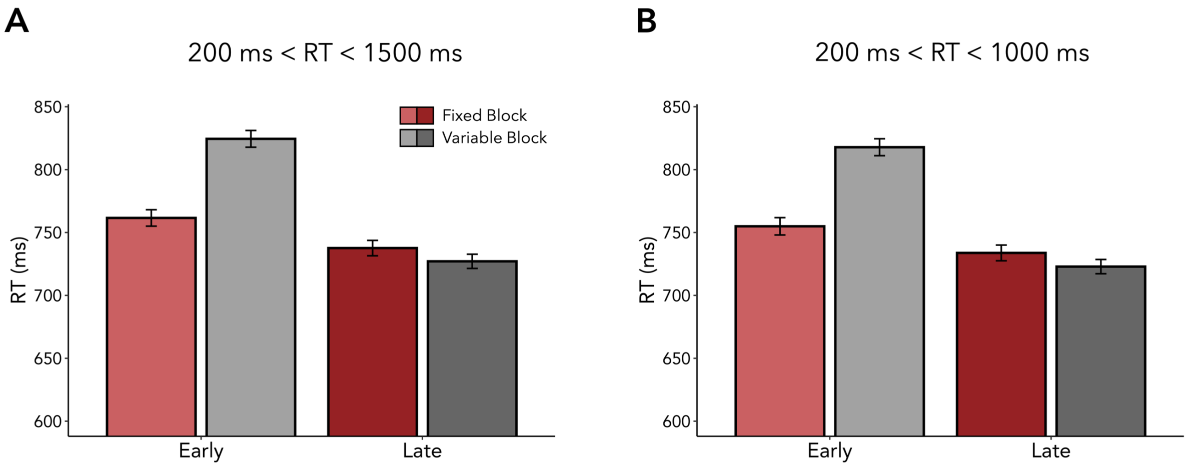

Supplementary Figure 1. Reaction time to the working-memory task after removing slow responses to the intervening task. Removing reaction times (RTs) to the intervening task that were faster than 200 ms and slower than 1500 ms (**A**, same as Figure 1B) or removing RTs that were faster than 200 ms and slower than 1000 ms (**B**) resulted in the same pattern of results. RTs to early memory probes were faster when the probe occurred in a fixed as opposed to a variable block, however, there was no difference in RTs to fixed-late versus variable-late probes. RTs to the memory probe were slower in variable-early than variable-late bocks, however, there was no difference in RTs to fixed-early versus fixed-late probes. Error bars indicate ± 1 standard error of the mean.

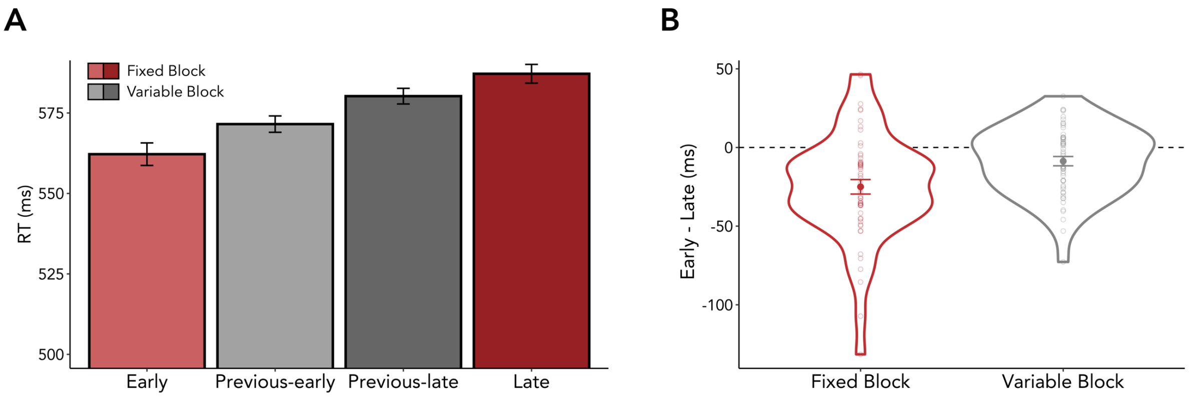

Supplementary Figure 2. Between-task task consequences in fixed and variable blocks. (A) Reaction times (RTs) to the intervening task in fixed and variable blocks. Variable-block trials are split into trials in which the previous delay condition was early versus late. **(B)** Violin plots depicting the difference in RTs between early and late trials in fixed blocks (Fixed-early – Fixed-late) and between previous early and previous late trials in variable blocks (Previous-early – Previous-late). This difference was significantly stronger in fixed versus variable blocks. Error bars indicate ± 1 standard error of the mean.

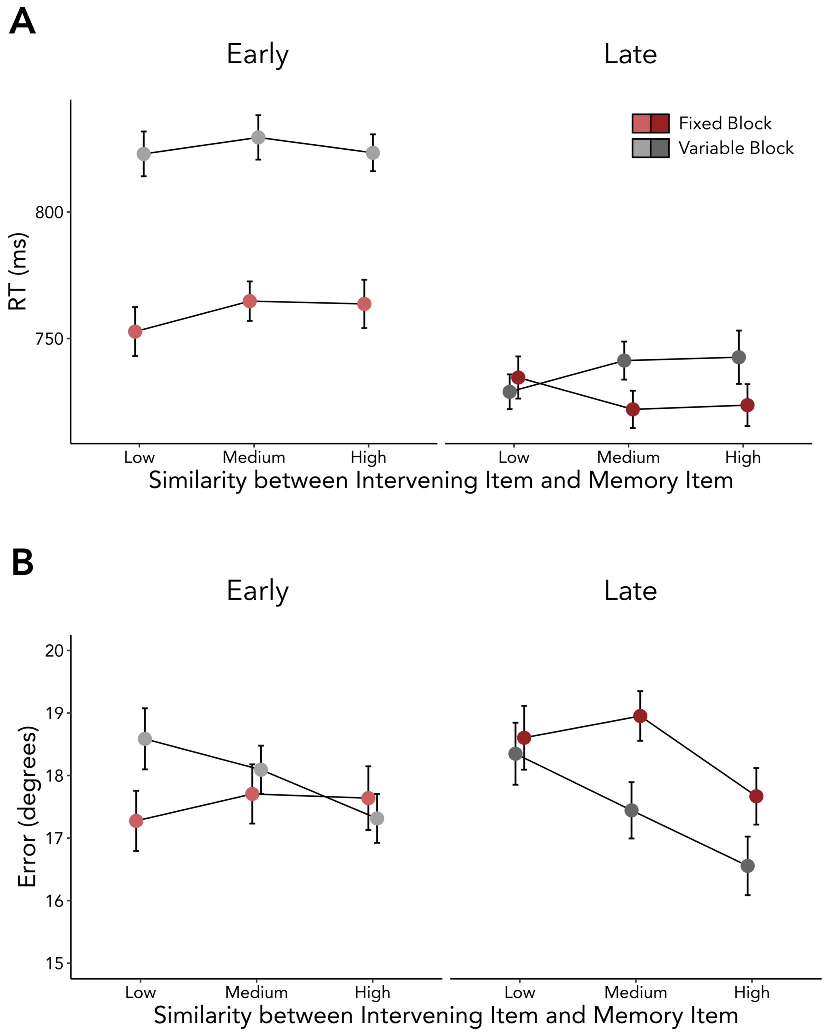

Supplementary Figure 3. Effect of sensory similarity on working-memory performance for early and late trials in fixed and variable blocks. (A) Reaction times (RTs) did not differ between different levels on sensory similarity. (B) Reproduction errors were the smaller when sensory similarity was high versus low and when sensory similarity was high versus medium. Error bars indicate ± 1 standard error of the mean.

Supplementary Table 1. Pairwise comparisons of reaction times (RTs) to the working-memory task between the different levels of delay condition and block type. The analysis was performed following pre-processing steps described in "Analysis", including the removal of RTs to the intervening task which were faster than 200 ms and slower than 1500 ms, as well as after removing RTs to the intervening task that were faster than 200 ms and slower than 1000 ms. This resulted on average in 4.83 ± 2.78% removed trials per participant for the former, and 8.42 ± 8.80% for the latter. Pairwise comparisons revealed the same pattern of results after removing RTs to the intervening task that were faster than 200 ms and slower than 1500 ms and after removing RTs to the intervening task that were faster than 200 ms and slower than 1000 ms.

	Trials removed	Fixed-early vs. Variable-early	Fixed-late vs. Variable-late	Fixed-early vs. Fixed-late	Variable-early- vs. Variable late
200 ms < intervening-task RT < 1500 ms	4.83 ± 2.78%	$t_{(53)} = -7.437$<br>$p_{Bonferroni} < 0.001$<br>$d = 1.012$	$t_{(53)} = 1.568$<br>$p_{Bonferroni} = 0.491$<br>$d = 0.213$	$t_{(53)} = 2.531$<br>$p_{Bonferroni} = 0.058$<br>$d = 0.344$	$t_{(53)} = 10.633$<br>$p_{Bonferroni} < 0.001$<br>$d = 1.447$
200 ms < intervening-task RT < 1000 ms	8.42 ± 8.80%	$t_{(53)} = -6.977$<br>$p_{Bonferroni} < 0.001$<br>$d = 0.950$	$t_{(53)} = 1.558$<br>$p_{Bonferroni} = 0.501$<br>$d = 0.212$	$t_{(53)} = 2.183$<br>$p_{Bonferroni} = 0.134$<br>$d = 0.297$	$t_{(53)} = 10.611$<br>$p_{Bonferroni} < 0.001$<br>$d = 1.444$

Supplementary Table 2. Main and interaction effects of reaction times (RTs) for the factors block type (fixed vs. variable), delay condition (early vs. late), and sensory similarity (low vs. medium vs. high), tested with a 2×2×3 repeated-measures ANOVA. This analysis yielded a significant main effect of block type and delay condition, and a significant interaction between these two factors. * indicates $p < 0.05$, ** indicates $p < 0.001$.

	df	*F*	*p*	η^2_G
Block Type **	1, 53	24.909	< 0.001	0.005
Delay Condition **	1, 53	61.691	< 0.001	0.023
Sensory Similarity	2, 106	0.237	0.789	< 0.001
Block Type × Delay Condition **	1, 53	51.199	< 0.001	0.009
Block Type × Sensory Similarity	2, 106	2.492	0.088	< 0.001
Delay Condition × Sensory Similarity	2, 106	0.555	0.576	< 0.001
Block Type × Delay Condition × Sensory Similarity	2, 106	0.544	0.582	< 0.001

Supplementary Table 3. Main and interaction effects of reproduction errors for the factors block type (fixed vs. variable), delay condition (early vs. late), and sensory similarity (low vs. medium vs. high), tested with a 2×2×3 repeated-measures ANOVA. This analysis yielded a significant main effect of block type and sensory similarity. * indicates p < 0.05, ** indicates p < 0.001.

	df	*F*	*p*	η^2_G
Block Type *	1, 53	5.340	0.025	0.002
Delay Condition	1, 53	0.291	0.592	< 0.001
Sensory Similarity *	2, 106	5.411	0.006	0.002
Block Type × Delay Condition	1, 53	0.596	0.443	< 0.001
Block Type × Sensory Similarity	2, 106	0.372	0.690	< 0.001
Delay Condition × Sensory Similarity	2, 106	1.360	0.261	< 0.001
Block Type × Delay Condition × Sensory Similarity	2, 106	2.562	0.082	0.001
